# The enigmatic wandering spleen: managing three cases in a tertiary setting

**DOI:** 10.1186/s12887-025-06005-9

**Published:** 2025-08-19

**Authors:** Mohammed Al Blooshi, Abdalla Aboelkheir, Avinash Hiremath, Ghadir Jaber, Mamoun AlMarzouqi

**Affiliations:** 1 Pediatric Surgery Division, Tawam Hospital, Al Ain, United Arab Emirates; 2 Department of Pediatric Surgery and Urology, Al Jalila Children’s Hospital, 6th Street, Al Jaddaf, Dubai, United Arab Emirates; 3 Department of Urology, Dubai Hospital, Dubai Health, Dubai, United Arab Emirates

**Keywords:** Wandering spleen, Pediatric, Splenic torsion, Splenectomy, Splenopexy

## Abstract

Wandering spleen is a rare clinical entity characterized by abnormal mobility of the spleen due to laxity or absence of its supporting ligaments. In pediatric patients, this condition can present with vague abdominal symptoms or acute torsion leading to infarction, posing a significant diagnostic challenge. Early identification through imaging and timely intervention are crucial to preserve splenic function. Conducted in a tertiary care setting, we report three pediatric cases of wandering spleen, each illustrating a different clinical course and surgical management strategy. The first case involved a 13-year-old girl with progressive abdominal pain and vomiting, ultimately requiring splenectomy after unsuccessful detorsion attempts. The second case featured a 10-year-old girl with a previously known pelvic spleen who underwent successful laparotomy and splenopexy upon finding a partially viable spleen. The third case involved a 3-year-old girl with fever, acute pain, and imaging evidence of an ectopic spleen; intraoperative detorsion and splenopexy preserved splenic tissue, underscoring the importance of salvage when possible. These cases highlight the variable presentation of wandering spleen, the pivotal role of imaging in diagnosis, and underscore that splenic salvage should be prioritized whenever feasible to maintain immunologic function. Splenopexy remains the preferred option for viable spleens, while splenectomy is reserved for nonviable organs, with adequate follow-up essential to monitor for potential postoperative complications.

## Introduction

Wandering spleen is a rare but significant clinical entity in which the spleen exhibits abnormal mobility within the abdomen due to lax or absent supporting ligaments [1]. Though precise epidemiological data remain limited, it is generally regarded as an uncommon condition in children, with some estimates suggesting it accounts for less than 2% of all splenic pathologies in the pediatric population [2]. Under normal circumstances, the spleen develops in the dorsal mesogastrium during embryogenesis and becomes anchored in the left upper quadrant by ligamentous attachments, including the gastrosplenic, splenorenal, and splenocolic ligaments [3]. When these ligaments fail to provide adequate support—whether from congenital anomalies or acquired weakening—the spleen can migrate freely, often descending into the lower abdomen or pelvis.

Although wandering spleen may remain asymptomatic for a prolonged period, its potential for torsion around the elongated vascular pedicle is a chief concern [4]. Torsion can compromise arterial and venous blood flow, resulting in congestion, ischemia, and, if untreated, splenic infarction. Pediatric patients can present a diagnostic challenge because their symptoms are frequently nonspecific, ranging from intermittent abdominal discomfort to severe pain with signs of acute abdomen. Studies suggest that approximately 56% of pediatric wandering spleen cases present with acute or intermittent torsion, underscoring the need for early detection [5].

Advanced imaging modalities have significantly improved the diagnostic process for this condition. Color Doppler ultrasound often serves as the first-line imaging technique, enabling rapid identification of an ectopic spleen and evaluating blood flow within the splenic vessels. In uncertain cases, contrast-enhanced computed tomography (CT) provides greater anatomical detail, identifying twisted pedicles or infarcted splenic tissue [6]. Timely diagnosis is crucial because management strategies differ markedly depending on splenic viability. When splenic perfusion is preserved or restored, splenopexy is preferred to maintain immunologic function and avoid the lifelong infection risks associated with asplenia. However, if the spleen is already infarcted, splenectomy becomes necessary [7]. In this paper, we present three pediatric cases that illustrate the diverse clinical and intraoperative findings associated with wandering spleen, illustrating the significance of individualized management and vigilant follow-up.

## Case presentation

Below, we detail three pediatric cases of wandering spleen. Each case emphasizes a unique clinical course, from chronic and progressive symptoms leading to splenectomy to more acute onsets where the spleen could be salvaged through splenopexy.

### Patient 1

#### Presentation

A previously healthy 13-year-old girl was referred to our service with a chief complaint of progressively worsening abdominal pain over three weeks. She also had intermittent vomiting, which became more frequent in the days preceding admission, and developed low-grade fever and lethargy over the final 24 h. On examination, she displayed tachycardia and appeared acutely unwell, with marked tenderness and guarding in the mid-abdomen. A palpable mass was noted, suggesting a possible organ displacement or enlargement. Notably, this patient had no history of trauma, surgeries, or connective tissue disorders, and her developmental history was unremarkable. Congenital anomalies of the supporting ligaments were suspected, although no specific associated conditions were identified.

#### Investigations

**Fig. 1:**
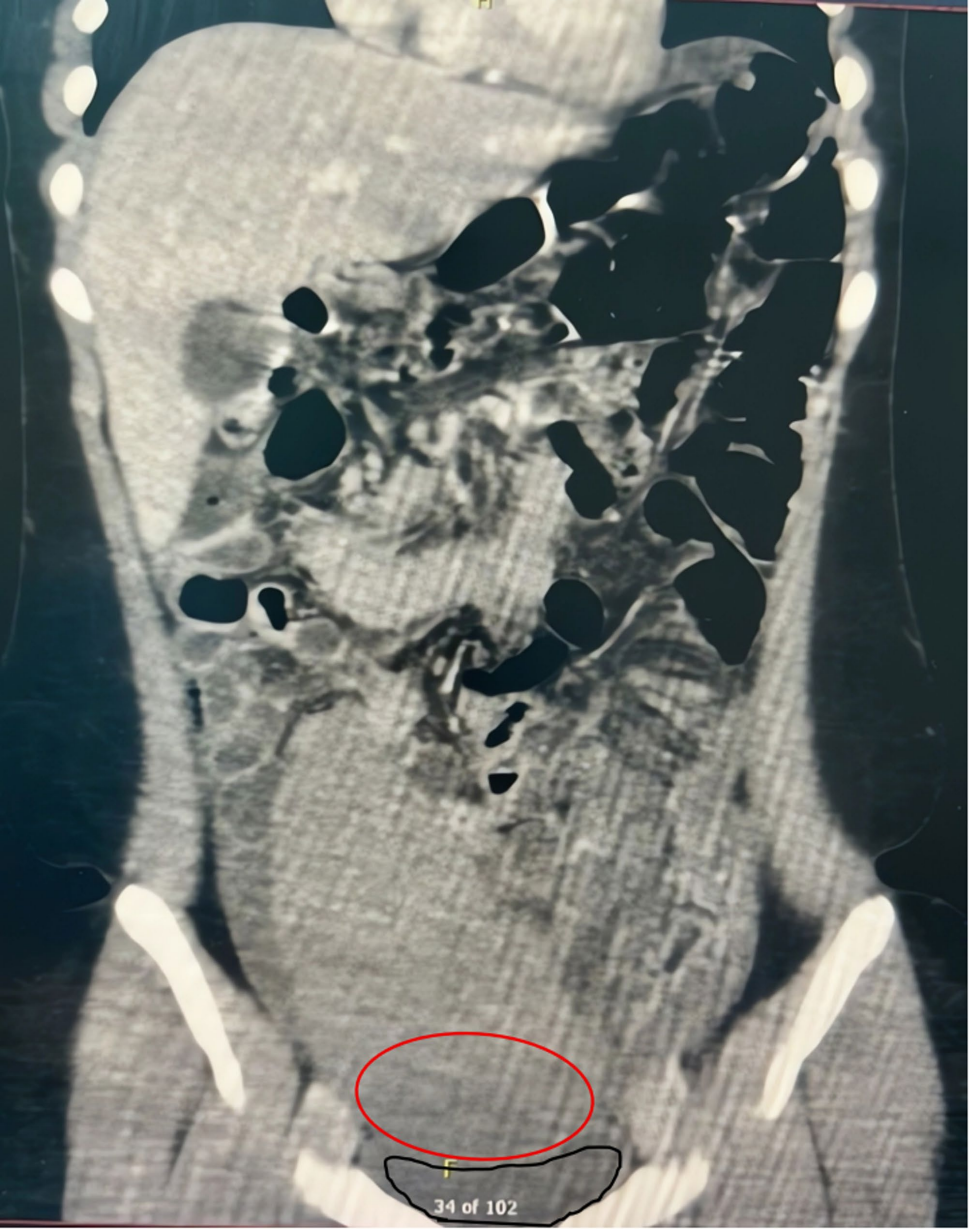
Intraoperative visualization of a twisted splenic pedicle. Operative photograph showing the completely twisted vascular pedicle and the congested spleen with dark discoloration, indicative of impaired blood flow. The red pen mark highlights the hypodense region representing splenic infarction, and the black pen mark denotes the urinary bladder compressed by the enlarged spleen

Laboratory tests revealed leukocytosis and elevated inflammatory markers, indicating an intra-abdominal inflammatory process. Initial ultrasound showed an abnormally positioned spleen with diminished Doppler flow. An abdominal Color Doppler ultrasound also revealed free intra-abdominal fluid and reduced flow to the ectopic spleen. A subsequent contrast-enhanced CT (Fig. 1) confirmed a significantly enlarged spleen outside its usual left upper quadrant location, with a twisted vascular pedicle consistent with splenic torsion; heterogeneous attenuation and absent enhancement suggested infarction. Given these findings, the differential diagnoses considered included ovarian torsion, mesenteric or omental cyst, mid-gut volvulus, splenic abscess, pancreatic pseudocyst, and complicated appendicitis.

#### Operative findings

**Fig. 2:**
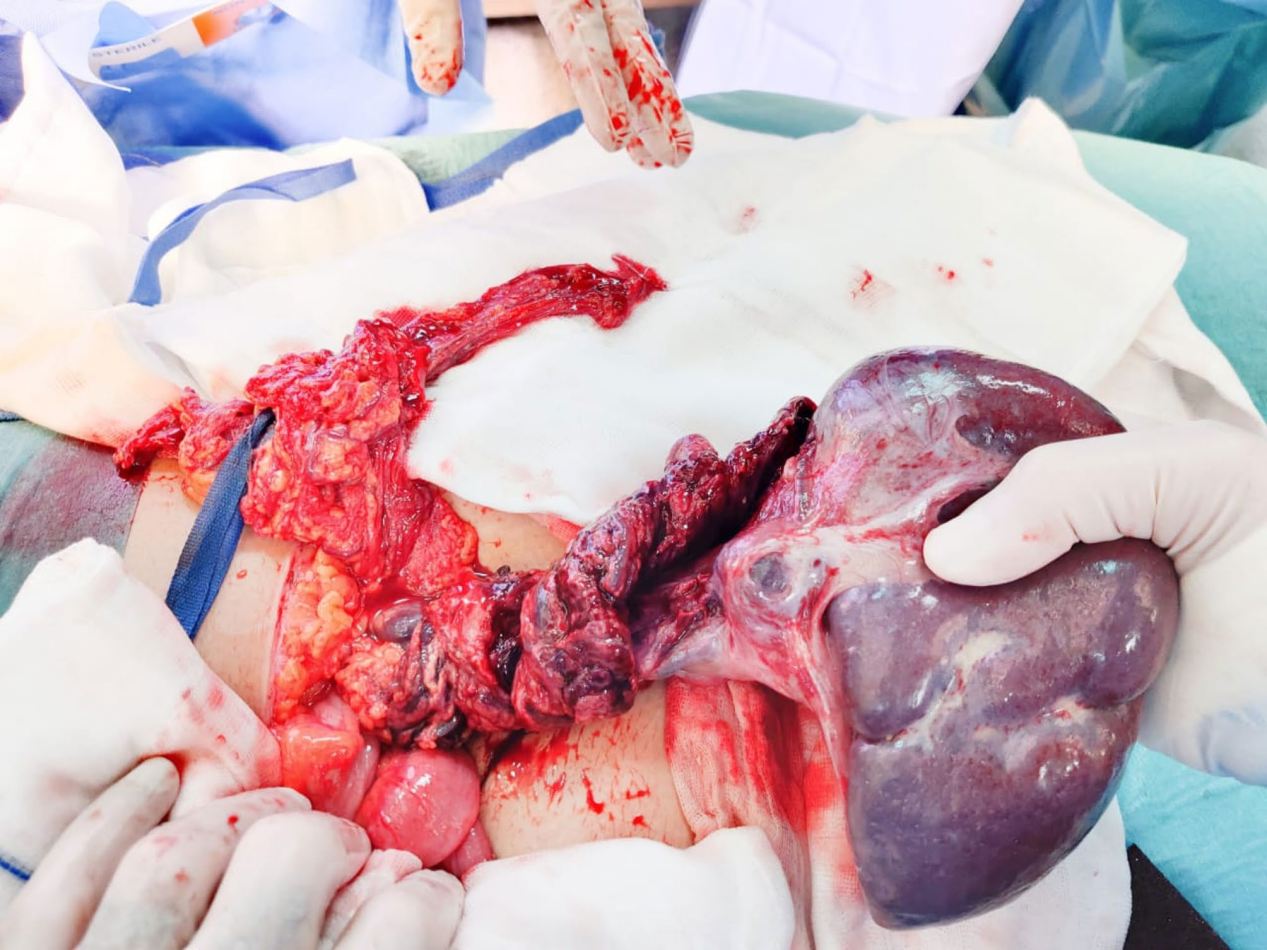
Intraoperative photograph illustrating torsion and infarction of a wandering spleen in patient 1

**Fig. 3:**
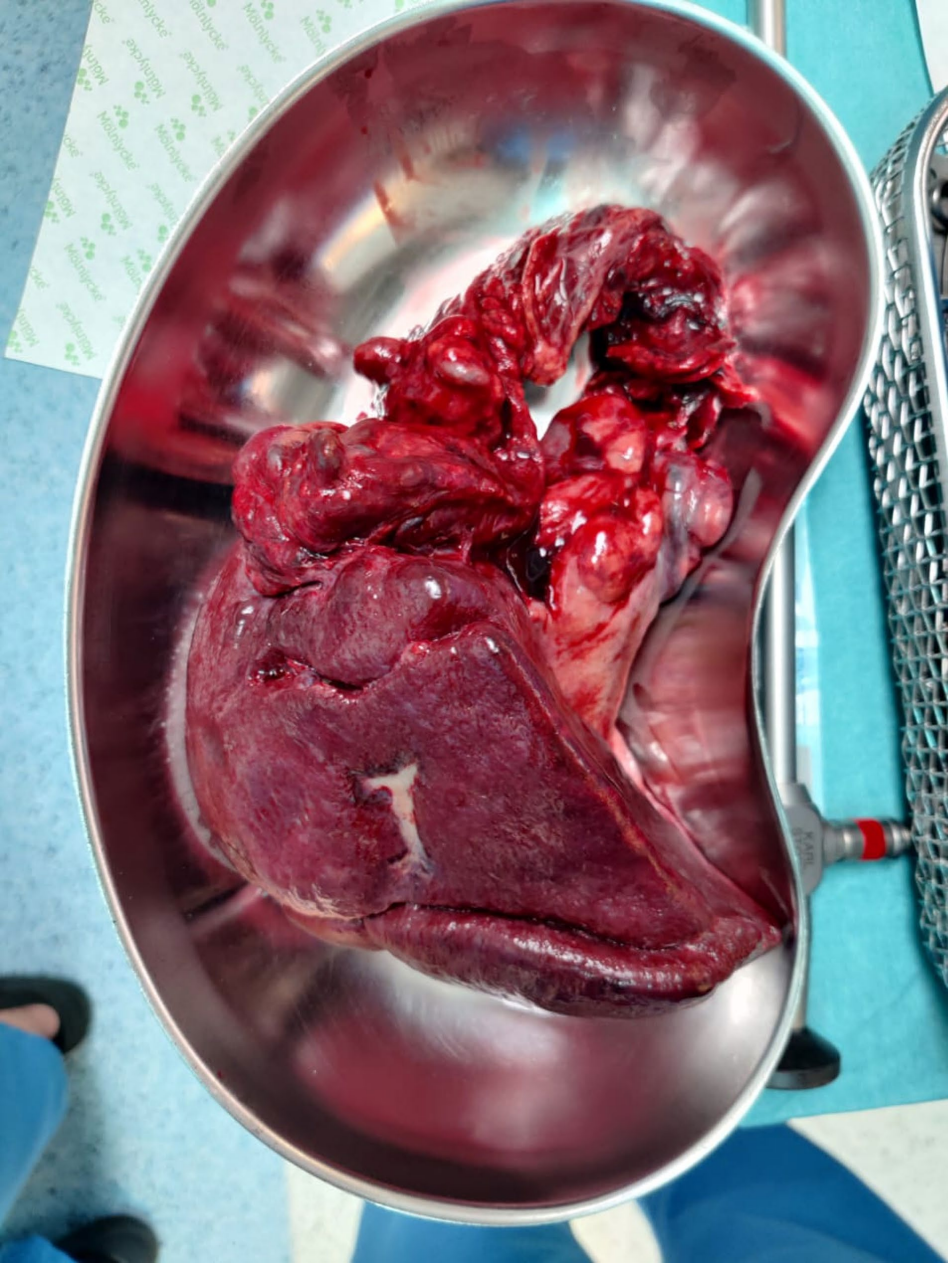
The resected spleen placed in a surgical basin, illustrating hemorrhagic infarction and loss of normal splenic architecture

Owing to these findings, she underwent an urgent exploratory laparotomy. Intraoperatively, the spleen was discovered to be twisted multiple times around its pedicle, with a dark, congested appearance suggesting nonviability. Detorsion, application of warm saline packs, and short-term observation did not restore adequate perfusion. Consequently, a splenectomy was performed to prevent sepsis or further hemodynamic compromise. She tolerated the procedure well. Figures 2 and 3 depict the intraoperative view of the twisted vascular pedicle and the resected spleen, confirming hemorrhagic infarction and loss of normal splenic architecture.

Fig. 2 Operative field after exploratory laparotomy showing an exteriorized ectopic (wandering) spleen with a twisted vascular pedicle (torsion). The spleen is diffusely dusky and congested with loss of normal lobulation, consistent with global infarction. 

#### Postoperative course and follow-up

At one month postoperatively, she returned for follow-up in the pediatric surgery clinic, reporting only mild, intermittent pain at the operative site. Physical examination revealed a well-healed surgical scar, and no further imaging was performed since the spleen had been removed. Histopathological analysis of the resected spleen showed expanded red pulp with focal hemorrhage, vascular proliferation, and areas of infarction. She was subsequently followed by the pediatric infectious disease (ID) team, receiving PCV20, meningococcal (ACWY and MenB) vaccines, and annual influenza vaccines, and was placed on prophylactic penicillin. She remains on periodic ID follow-up for asplenia management. Overall, her recovery was uneventful, with full resolution of symptoms.

### Patient 2

#### Presentation

A 10-year-old girl presented with a three-day history of moderate abdominal pain radiating to the back, accompanied by recurrent vomiting. She had been previously diagnosed with an ectopic or “pelvic” spleen during imaging for an unrelated issue. On examination, she was hemodynamically stable, reporting mild, diffuse abdominal tenderness, particularly in the lower quadrants. This patient had no significant past medical or surgical history, no evidence of connective tissue disease, and normal growth and development, suggesting a congenital etiology for her wandering spleen.

#### Investigations

An ultrasound reaffirmed the spleen’s pelvic location and revealed a “whirlpool” sign around the splenic vessels, highly suggestive of torsion. Differential considerations for a pelvic mass in this pre-adolescent girl included ovarian torsion, tubo-ovarian abscess, ectopic kidney, mesenteric cyst, and torsion of a wandering spleen.

#### Operative findings

**Fig. 4:**
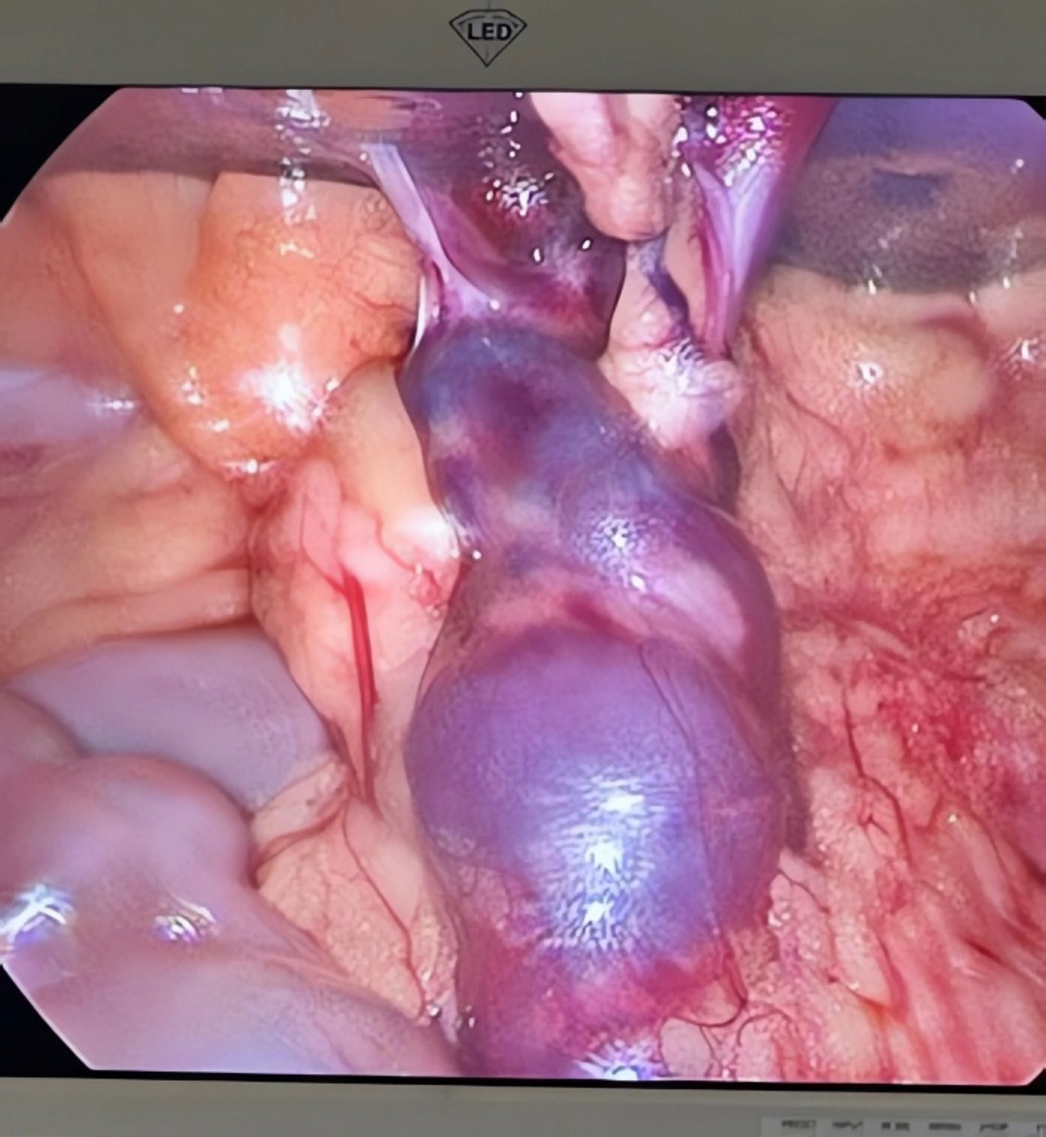
Laparoscopic view of the ectopic spleen twisted around its pedicle

**Fig. 5:**
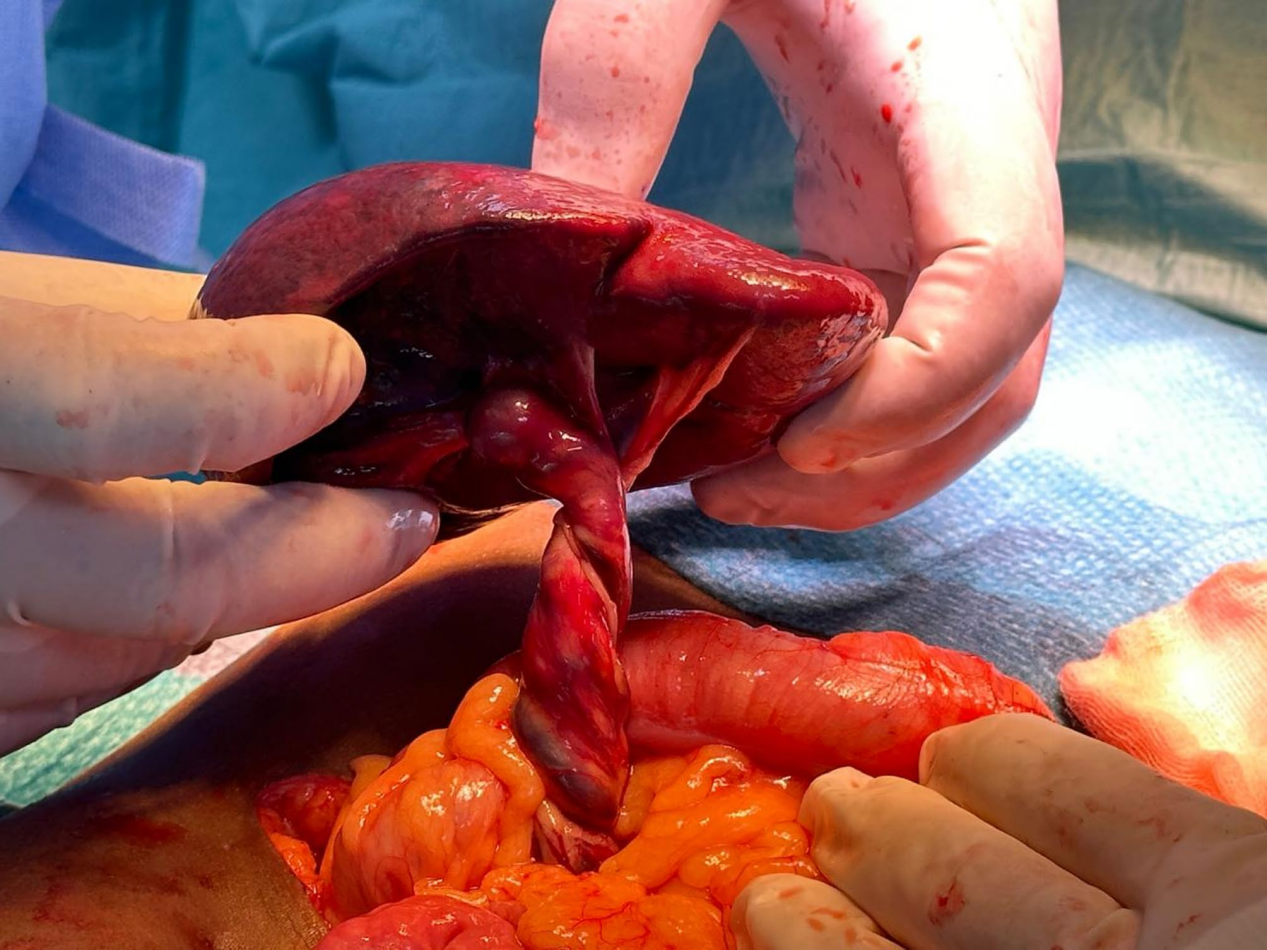
Intraoperative view of the spleen after detorsion, displaying partial congestion and the twisted vascular pedicle

**Fig. 6:**
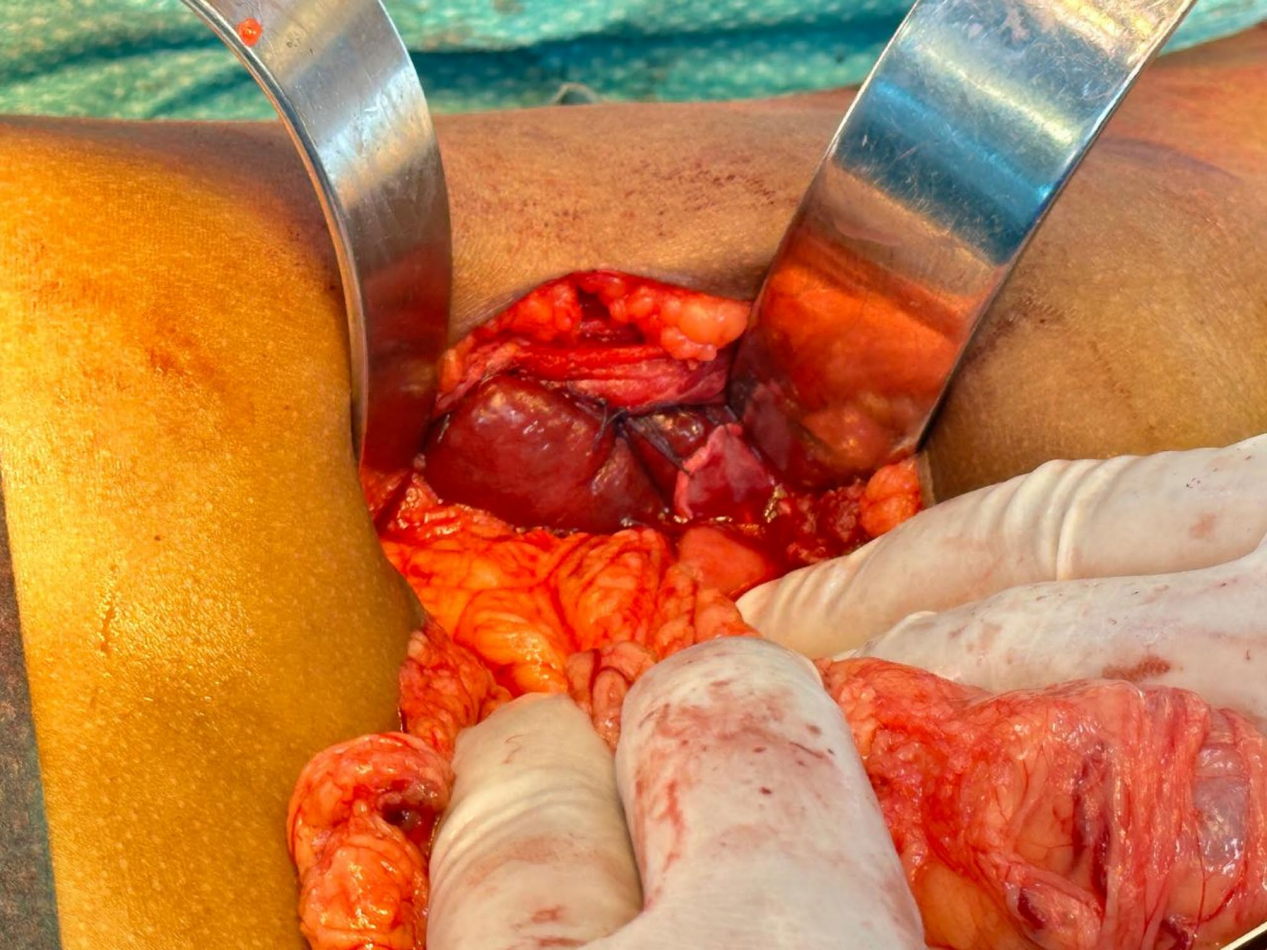
Additional intraoperative view showing the spleen being positioned and secured during splenopexy

Although a laparoscopic approach was initially attempted, limited operative fields and the considerable size of the ectopic spleen led to conversion to an open surgical technique (Fig. 4). Conversion was achieved via a left supraumbilical incision. Intraoperatively, the spleen was found to have undergone four complete rotations around its vascular pedicle, yet partial perfusion remained. After careful detorsion, partial viability was noted with the return of some splenic coloration and perfusion. The surgical team opted for splenopexy rather than splenectomy, anchoring the spleen to the left lateral abdominal wall with nonabsorbable sutures (Figs. 5 and 6).

#### Postoperative course and follow-up

Postoperative imaging confirmed stable perfusion, and the patient recovered without complications. Follow-up ultrasounds were performed at one month and six months post-surgery. At one month, the spleen was located in the left lumbar region with homogeneous echotexture, a small rim of fluid near the splenic hilum (attributed to postoperative changes), and no sign of re-torsion. At six months, she remained asymptomatic, and repeat ultrasound again confirmed stable splenic perfusion with no evidence of vascular pedicle twisting. Physical examination revealed the spleen palpable in the left lumbar region but otherwise unremarkable. She continues to do well with no laboratory-based monitoring required, and no further imaging abnormalities have been noted.

### Patient 3

#### Presentation

A 3-year-old girl arrived in the emergency department with a sudden onset of abdominal pain, fever, and vomiting that began one day prior. Her parents reported unusual lethargy and irritability. On examination, she was tachycardic, febrile, and had a distended abdomen with notable guarding. A firm, mobile mass was palpable in the periumbilical region extending toward the left flank, suggesting organ displacement. Further review of this patient’s history showed no prior surgeries, trauma, or systemic illnesses, and she had been growing normally with no developmental concerns.

#### Investigations

**Fig. 7:**
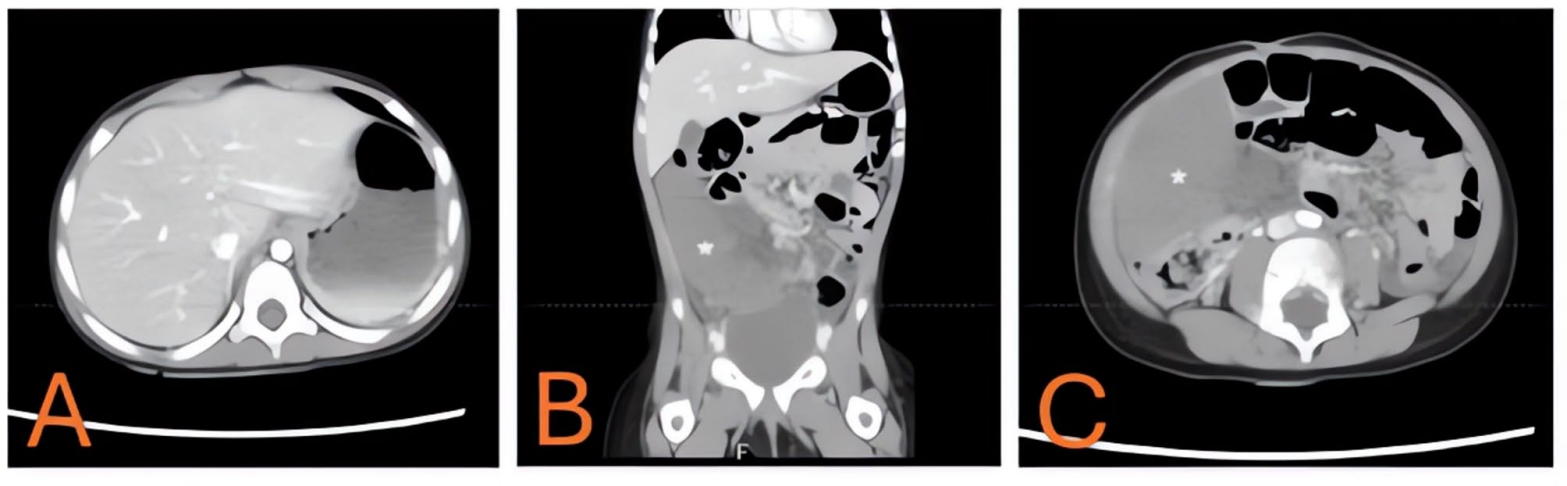
Contrast-enhanced CT illustrating ectopic spleen in a child with wandering spleen. **A** Axial CT slice at the level of the stomach shows no recognizable splenic silhouette in the left upper quadrant, suggesting ectopic displacement of the spleen. **B** Coronal CT demonstrating the spleen shifted to the mid-abdominal or right-sided location. **C** Axial CT again illustrating the ectopic spleen with heterogeneous attenuation

**Fig. 8:**
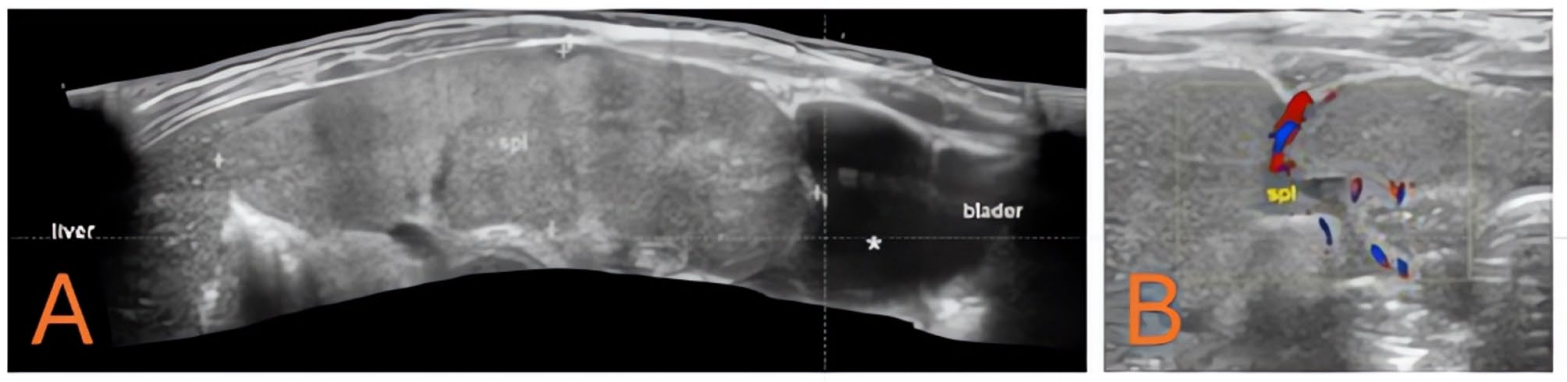
Panoramic ultrasound and color doppler of the ectopic spleen. **A** Panoramic ultrasound revealing the displaced spleen (spl) pressing against the bladder. **B** Color Doppler study showing reduced flow signals in the splenic vessels

Laboratory results demonstrated marked leukocytosis, mild anemia, and elevated inflammatory markers. An ultrasound revealed an ectopic spleen lying transversely in the abdomen with reduced vascular signals on color Doppler, consistent with torsion. A contrast-enhanced CT (Fig. 7) and an ultrasound (Fig. 8) confirmed a twisted pedicle and heterogeneous splenic parenchyma, raising concern for impending infarction. On initial presentation, her vital signs included a pulse rate of 134 beats per minute, blood pressure of 110/60 mmHg, respiratory rate of 28 breaths per minute, oxygen saturation of 98%, and a temperature of 38.1 °C. Laboratory tests revealed a WBC of 18.6 × 10^3/µL, hemoglobin of 8.9 µg/dL, and a CRP of 180.2 mg/L, consistent with an acute inflammatory process. The differential diagnoses considered in this toddler included intussusception, mid-gut volvulus, renal or adrenal mass, splenic abscess, and torsion of a wandering spleen.

#### Operative findings

She underwent an emergent laparotomy, which confirmed multiple twists of the splenic pedicle. Detorsion and application of warm saline packs restored partial reperfusion, prompting the surgical team to perform a splenopexy. A retroperitoneal pouch was fashioned to secure the spleen and minimize recurrence.

#### Postoperative course and follow-up

The child’s postoperative course was uneventful; subsequent imaging and clinical examinations demonstrated preserved splenic function. One month after surgery, she returned for a follow-up visit, reporting no abdominal pain or constitutional symptoms. Ultrasound performed at that time showed a stable spleen position with improved perfusion, although the exact imaging records are no longer available. Unfortunately, the family was lost to further follow-up, limiting long-term outcome data. Nonetheless, at the last known evaluation, she had maintained normal growth parameters and remained asymptomatic.

Table 1 provides a concise overview of the cases, focusing on clinical presentation, imaging findings, and surgical approaches.

**Table 1:** Summary of clinical presentation, imaging findings, and surgical management

Patient	Gender & age	Clinical presentation	Physical examination	Ultrasound findings	CT findings	Surgery
1	F, 13	3-week abdominal pain	Tense abdomen, large palpable mid-abdominal mass	Ectopic spleen, diminished Doppler flow	Enlarged spleen with pedicle torsion and infarction	Splenectomy
2	F, 10	3-day abdominal pain	Pelvic mass; mild tenderness	Pelvic spleen; “whirlpool” sign suggestive of torsion	Twisted pedicle with heterogeneous attenuation suggestive of evolving infarction	Splenopexy with lateral-wall suture fixation (non-absorbable sutures)
3	F, 3	1-day abdominal pain, fever	Tense abdomen; mass extending to the left	Enlarged, transversely oriented spleen; reduced flow	Heterogeneous attenuation indicative of possible infarction	Splenopexy with retroperitoneal pouch

## Discussion

Wandering spleen in pediatric patients demonstrates the interplay between a rare anatomical anomaly and potentially severe outcomes. Though its exact incidence remains elusive, it may represent less than 2% of all splenic pathologies in children [2], highlighting both its rarity and the necessity for heightened clinical awareness. Embryologically, the spleen originates in the dorsal mesogastrium, and failure or weakening of the ligamentous attachments can predispose it to migrate within the abdominal cavity, often descending into the lower abdomen or pelvis. In particular, congenital absence or elongation of these supporting ligaments—such as the gastrosplenic, splenorenal, and phrenicocolic ligaments—allows excessive mobility of the spleen and increases the risk of torsion. Notably, up to two-thirds of pediatric wandering spleen cases may present with acute or intermittent torsion [5], and these children can exhibit vague gastrointestinal complaints or severe abdominal pain, highlighting the diagnostic challenge. When the spleen migrates from its usual position, the vascular pedicle may twist, threatening blood supply and splenic viability.

Early recognition is critical, as children can present either with nonspecific abdominal discomfort or more alarming signs of acute abdomen and instability [8]. In such situations, Doppler ultrasound is typically the first-line investigation, revealing abnormal splenic positioning and reduced blood flow, while contrast-enhanced CT offers detail on the extent of torsion and possible infarction [9]. Once torsion occurs, compromised arterial and venous flow can lead to congestion, ischemia, and eventual infarction if not promptly addressed. Our series further emphasizes that timely imaging not only delineates splenic malposition but also guides the decision between splenopexy—if perfusion is restorable— or splenectomy, as was necessary in Patient 1, when irreversible infarction has set in. In our series, the need for splenectomy in Patient 1 versus the successful splenopexies in Patients 2 and 3 exemplifies how prompt imaging-guided intervention can shift management from organ loss to organ preservation, thereby avoiding the lifelong infectious risk of asplenia.

The surgical approach depends on spleen viability. If perfusion can be restored, splenopexy—via suture fixation or retroperitoneal pouch—is preferred to preserve immune function. Additional pediatric techniques such as mesh “hammocks,” omental slings, and totally laparoscopic extraperitoneal pouches have also been reported, each conferring > 90% torsion-free survival while maintaining normal splenic immunologic parameters [10]. In contrast, extensive infarction necessitates splenectomy, as demonstrated in Patient 1.

Laparoscopic splenopexy is favored for its minimally invasive benefits, although conversion to open surgery may be required in cases of significant splenic enlargement or complex adhesions. A growing body of pediatric literature supports these observations. Patterson et al. recently reported a 12-year-old girl treated with mesh-assisted laparoscopic splenopexy, with durable splenic perfusion on follow-up, paralleling the favorable outcomes of our Patients 2 and 3 [9]. Shakoor and colleagues later described a 10-year-old child treated with mesh-assisted pre-peritoneal splenopexy, underscoring the technical versatility—and safety—of minimally invasive organ-preservation techniques [11]. In contrast, Melkamu et al. detailed a 16-year-old girl who required emergency splenectomy for gangrenous torsion after delayed presentation, reinforcing that time-to-diagnosis remains the decisive factor dictating salvage versus removal [12]. Juxtaposing these external reports with our series strengthens the premise that prompt imaging-guided intervention markedly improves splenic salvage rates, whereas prolonged ischemia invariably necessitates splenectomy.

Moreover, in our practice, we often attempt a laparoscopic approach first; however, if the spleen is massively enlarged—requiring a large incision for extraction anyway—or if the patient’s clinical condition calls for an immediate and more extensive exploration, an open surgical approach becomes more appropriate [13].

The ultimate decision is primarily influenced by the surgical team’s collective experience with minimally invasive techniques. High-volume pediatric centers with established laparoscopic programs achieve higher splenic-salvage rates and shorter operative times, whereas centers with limited experience tend to convert early or default to open surgery, increasing morbidity. Greater experience refines intraoperative judgment—recognizing subtle colour change after detorsion or assessing Doppler flow—which directly guides salvage versus removal decisions. Multidisciplinary familiarity with postoperative tools such as point-of-care ultrasound to confirm reperfusion streamlines care, and surgeon preference plus operative expertise ultimately determine whether laparoscopy or laparotomy is selected.

Post-splenectomy, children require vaccination against encapsulated organisms to reduce the risk of overwhelming infection [14]. In emergency situations, we follow the accelerated schedule endorsed by ACIP [15]: once the child is clinically stable (preferably ≥ 14 days post-operatively), administer a pneumococcal conjugate vaccine (PCV15 or PCV20); if PCV15 is used, give PPSV23 eight weeks later. A single Haemophilus influenzae type b (Hib) dose is provided if age-appropriate protection is uncertain. The first quadrivalent meningococcal conjugate vaccine (MenACWY) dose is delivered before discharge, with boosters every five years, and a serogroup B meningococcal vaccine series is initiated (B-fbx at 0 and 6 months or B-4c at 0, 1–2, and 6 months). Annual inactivated influenza vaccination completes the regimen.

Current ACIP guidance further recommends age-appropriate PCV15/PCV20 followed by PPSV23 boosters, quadrivalent (MenACWY) and serogroup B (MenB) meningococcal vaccines, Haemophilus influenzae type b, and annual influenza immunization, with periodic antibody-titer or pitted-red-cell monitoring to confirm protection [15]. Where antibody surveillance is unavailable, daily penicillin V prophylaxis until at least age 16 years or for a minimum of two years post-splenectomy remains standard practice [16]. In our Patient 1, who underwent splenectomy, early postoperative follow-ups with both pediatric surgery and infectious disease teams confirmed adherence to these guidelines, with routine revaccination and prophylactic penicillin. Laparoscopic splenopexy is favored for its minimally invasive benefits, although conversion to open surgery may be required in cases of significant splenic enlargement or complex adhesions.

## Conclusion

Wandering spleen is an uncommon yet significant cause of pediatric abdominal pain. Prompt clinical suspicion and imaging differentiate it from more routine causes. Treatment—splenopexy or splenectomy—hinges on splenic viability. Salvage via splenopexy preserves immunologic function, whereas splenectomy demands vigilant immunizations and follow-up. These considerations ensure the best prognosis in a challenging but important clinical condition.

## Data Availability

The datasets generated during and/or analyzed during the current study contain confidential patient information and are not publicly available. De-identified data may be shared by the corresponding author on reasonable request and with prior Institutional Review Board approval.
